# The important role of circulating CYFRA21-1 in metastasis diagnosis and prognostic value compared with carcinoembryonic antigen and neuron-specific enolase in lung cancer patients

**DOI:** 10.1186/s12885-017-3070-6

**Published:** 2017-02-02

**Authors:** Li Zhang, Dan Liu, Lei Li, Dan Pu, Ping Zhou, Yuting Jing, He Yu, Yanwen Wang, Yihan Zhu, Yanqi He, Yalun Li, Shuang Zhao, Zhixin Qiu, Weimin Li

**Affiliations:** 1 Department of Respiratory Medicine, West China Hospital, Sichuan University, Chengdu, Sichuan Province 610041 People’s Republic of China; 2 Lab of Pathology, West China Hospital, Sichuan University, Chengdu, Sichuan Province 610041 People’s Republic of China; 3 Clinic Skill Center, West China Hospital, Sichuan University, Chengdu, Sichuan Province 610041 People’s Republic of China

**Keywords:** Lung cancer patients, Biomarkers, CYFRA21-1, CEA, NSE, Metastasis, Prognosis

## Abstract

**Background:**

The roles of carcinoembryonic antigen (CEA), cytokeratin 19 fragments (CYFRA21-1) and neuron-specific enolase (NSE) in metastases occurrence and poor diagnosis in specific histological classifications of lung cancer need further exploring. In this study, we investigated relationship between elevated levels of three biomarkers of CEA, CYFRA21-1 and NSE (individually and in combination) and metastasis, survival status and prognosis in lung cancer patients.

**Methods:**

Eight hundred and sixty eight lung cancer patients including adenocarcinoma (ADC, *N* = 445), squamous cell carcinoma (SCC, *N* = 215), small cell lung cancer (SCLC, *N* = 159) and other types (*N* = 49) were categorized into negative, moderate and high groups according to serum levels of biomarkers, and were then categorized into negative, single, double and triple groups according to any positive combination of three biomarkers. The cutoff values of three biomarkers for groupings were developed on the training group (*N* = 432) and verified in a validation group (*N* = 436). Clinical and laboratory characteristics were then assessed for correlation with occurrence of metastasis, survival status and prognosis between the two groups. Further correlation analyses were also conducted by different subtypes (ADC, SCC and SCLC) and tumor stages (I + II, III and IV) of lung cancers.

**Results:**

The consistent results between training and validation group confirmed the rationality of grouping methods. CYFRA21-1 levels had stronger association with metastases and survival status than CEA and NSE in all lung cancer patients. When stratified by subtypes, these significances only existed in ADC patients for CYFRA21-1. Cox regression analyses showed that CYFRA21-1 and NSE were independent prognostic factors for lung cancer patients. However, only CYFRA21-1 was an independent prognostic factor in ADC and SCLC patients subtypes. Cox-regression results also indicated that CYFRA21-1 could act as independent prognostic factor in different stages (I + II, III and IV) of lung cancer.

**Conclusion:**

CYFRA21-1 was more important in metastasis occurrence and in predicting poor prognosis in lung cancer patients than CEA, NSE and positive numbers of biomarkers.

**Electronic supplementary material:**

The online version of this article (doi:10.1186/s12885-017-3070-6) contains supplementary material, which is available to authorized users.

## Background

Globally, lung cancer has the highest associated mortality among all malignant cancers [1]. The 5-year survival rate in advanced stage cancers is 15%, as compared to 80% in early stage lung cancers [2]. One of the reasons is that most patients are diagnosed at advanced stages due to lack of sensitive and specific early diagnostic biomarkers [3]. Non-small-cell lung cancer (NSCLC) accounts for approximately 85% of all lung cancers; the remaining 15% cases are classified as small cell lung cancer (SCLC) [4]. Although chemotherapy and targeted therapy are the main clinical treatment especially of stage IV patients, yet there is only 4–5% improvement in 5-year survival rates for stage I-III patients, and no significant improvement for stage IV patients [5]. The diagnostic methods include chest x-ray, computed tomography (CT) and needle biopsy of lung [6, 7]. However, the high cost and/or invasive nature of these investigations limit the widely use in clinical diagnosis.

During past decades, many advances have been made in the identification of tumor-associated markers in body fluids such as plasma, serum or bio-aerosols such as exhaled breath condensate (EBC) [8, 9]which could facilitate early detection and help for treatment monitoring [10]. For lung cancer diagnosis, the leading markers used are carcinoembryonic antigen (CEA), cytokeratin 19 fragments (CYFRA 21–1) and neuron-specific enolase (NSE). CEA, which is closely related to histological classification, is considered valuable for diagnosis of ADC [11]. CYFRA 21–1 and NSE are used for the diagnosis of SCLC [12, 13]. Increasing trend in levels of CEA, CYFRA21-1 and NSE have been associated with metastasis and poor prognosis [14–16]. However, limitations of previous studies are either in small sample sizes (*N* = 200-300) or not analyzed in combinations.

In this retrospective study we evaluated the predictive values of serum levels of CEA, CYFRA21-1 and NSE for prognosis and occurrence of metastasis, and the association of these biomarkers with clinical characteristics.

## Methods

### Patients

This study recruited 868 lung cancer patients who were admitted to West China Hospital between 2008 through 2012. All data were obtained from medical records within 2 weeks of diagnosis, and information regarding metastasis was obtained through reports of whole-body CT scan, bone scan, lymph node and fiber optic bronchoscopy biopsy. Survival time was obtained during subsequent follow-up visit or telephonic inquiry. Those patients who did not receive CEA, CYFRA21-1 and NSE determinations and lack of follow-up data were excluded. Data on stage were according to the TNM Classification of Malignant Tumors, 7th Edition [17].

The overall survival time was calculated as time from the date of diagnosis through the date of death or last follow up visit (if the exact date of death was unavailable). Prior to surgery or any other treatments, serum concentrations of CEA, CYFRA21-1 and NSE were measured by immunoassays. Based on the reported literatures, the threshold values for CEA, CYFRA21-1 and NSE levels were 3.4 ng/mL, 3.0 ng/mL and 15.0 ng/mL, respectively [17].

### Study design

Depending on the levels of CEA, CYFRA21-1 and NSE, the study subjects were divided into three groups (negative, moderate and high). For CEA analysis, moderate and high groups were defined as 1–10 folds and >10 folds cutoff value, respectively. For CYFRA21-1 analysis 1–3 folds and >3 folds, respectively. For NSE analysis, 1–2 folds and >2 folds, respectively. This analysis was performed in a randomly selected training group (*N* = 432), reserving the left 436 cases for validation. The cutoff values of three biomarkers for groupings were developed on the training group and tested in a validation group.

Next, we determined the correlations of biomarker levels with three main histological subtypes, ADC, SCC and SCLC. The association analyses of other tumor types (*N* = 49) such as large cell lung cancers and adenosquamous carcinoma were also performed which showed no positive prognostic value (Data not shown).

Finally, the diagnosis, metastasis and prognostic values of combination patterns of three biomarkers were also evaluated. In brief, patients were grouped as negative, single, double and triple positive of biomarkers. Negative indicated that serum levels of all three biomarkers were below cutoff levels. Single, double, triple positive meant that concentrations of any one, two or all three biomarkers exceeded cutoff values.

### Statistical methods

SPSS 19.0 for Windows (SPSS Inc, Chicago, USA) was used for data analyses. Chi-square test was performed to evaluate the inter-group differences. Kaplan-Meier test was used to calculate the survival status of different groups, and Log-rank test was used to compare the survival among three groups. Multivariate Cox regression analysis was used to determine the clinical characteristics, metastasis and survival status in order to estimate the hazards ratio for different serum levels of CEA, CYFRA21-1 and NSE and identify independent predictors of poor prognosis.

## Results

### Increased levels of CYFRA21-1 significantly correlated with metastatic disease

Total 868 lung cancer patients were randomly divided into training group (TA, 432 cases) and validation (VA, 436 cases) group to confirm the rationality of grouping methods. Among them, 320 patients tested negative (TA: 164, VA: 156) (<3.4 ng/mL) while 365 (TA: 179, VA: 186) and 210 (TA: 89, VA: 94) had moderate and high levels of CEA, respectively. For CYFRA21-1, 231 patients tested negative (TA: 115, VA: 116) while 390 (TA: 190, VA: 200) and 247 (TA: 127, VA: 120) had moderate and high levels, respectively. For NSE, 412 patients (TA: 206, VA: 206) tested negative while 256 (TA: 128, VA 128) and 200 (TA: 98, VA: 102) had moderate and high levels.

The results indicated strong correlations of increased levels of CEA, CYFRA21-1 and NSE with histological classifications in both TA and VA groups (All *P* < 0.001). CEA and CYFRA21-1 were also related closely to TNM stages in TA and VA groups (*P* < 0.05, *P* < 0.01 and *P* < 0.001), while NSE had dramatic correlation with smoke status (TA: *P* < 0.01, VA: *P* < 0.05). CEA correlated closely to bone metastasis (TA: *P* < 0.05, VA: *P* < 0.01) and NSE had significant correlation with metastasis of bone (TA: *P* < 0.001, VA: *P* < 0.01), liver (TA: *P* < 0.001, VA: *P* < 0.01), lymph node (TA: *P* < 0.01, VA: *P* < 0.01) and mediastinum (TA: *P* < 0.01, VA: *P* < 0.05) (Table 1, Additional file 1: Table S1A and B).

**Table 1:** The analysis of CYFRA21-1 in all lung cancer patients

	No. (%)				
	Neg	Moderate	High	Total	*P* Value
		1–3 fold	>3 fold		
	(*n* = 115)	(*n* = 190)	(*n* = 127)	(*n* = 432)	
Basic Characteristics					
Age					
< 45	5 (4.3)	16 (8.4)	7 (5.5)	28	0.101
45–60	58 (50.4)	72 (37.9)	46 (36.2)	176	
> 60	52 (45.3)	102 (53.7)	74 (58.3)	228	
Sex					
Male	79 (68.7)	134 (70.5)	83 (65.4)	296	0.623
Female	36 (31.3)	56 (29.5)	44 (34.6)	136	
Histological classification					
SCC	21 (18.3)	42 (22.1)	56 (44.1)	119	
ADC	64 (55.7)	103 (54.2)	58 (45.7)	225	<0.001**
SCLC	22 (19.1)	34 (17.9)	7 (5.5)	63	
Others	8 (6.9)	11 (5.8)	6 (4.7)	25	
Stages					
I	12 (10.4)	6 (3.2)	0 (0.0)	18	<0.001**
II	7 (6.1)	8 (4.2)	2 (1.6)	17	
III	40 (34.8)	50 (26.3)	38 (29.9)	128	
IV	49 (42.6)	115 (60.5)	79 (62.2)	243	
#Un.	7 (6.1)	11 (5.8)	8 (6.3)	26	
Smoke status					
No	55 (47.8)	80 (42.1)	56 (44.1)	191	0.621
Yes	60 (52.2)	110 (57.9)	71 (55.9)	241	
Metastasis					
Brain					
No	104 (90.4)	162 (85.3)	105 (82.7)	371	0.212
Yes	11 (9.6)	28 (14.7)	22 (17.3)	61	
Bone					
No	101 (90.4)	142 (74.7)	92 (72.4)	335	<0.01**
Yes	14 (9.6)	48 (25.3)	35 (27.6)	97	
Liver					
No	113 (98.3)	170 (89.5)	108 (85.0)	391	<0.01**
Yes	2 (1.7)	20 (10.5)	19 (15.0)	41	
Adrenal gland					
No	113 (98.3)	173 (91.1)	119 (93.7)	405	<0.05*
Yes	2 (1.7)	17 (8.9)	8 (6.3)	27	
Lymph node					
No	66 (57.4)	68 (35.8)	37 (29.1)	171	<0.001***
Yes	49 (42.6)	122 (64.2)	90 (70.9)	261	
Intrapulmonary					
No	105 (91.3)	163 (85.8)	111 (87.4)	379	0.360
Yes	21 (9.1)	58 (14.9)	35 (14.2)	53	
Pleural					
No	100 (87.0)	170 (89.5)	98 (77.2)	368	<0.01**
Yes	15 (13.0)	20 (10.5)	29 (22.8)	64	
Mediastinal					
No	113 (98.3)	185 (97.4)	120 (94.5)	418	0.208
Yes	2 (1.7)	5 (2.6)	7 (5.5)	14	
Peritoneum					
No	115 (100)	178 (93.7)	116 (91.3)	409	<0.01**
Yes	0 (0.0)	12 (6.3)	11 (8.7)	23	
Validation group					
No. (%)					
Neg	Moderate	High	Total	*P* Value	
	1–3 fold	>3 fold			
(*n* = 116)	(*n* = 200)	(*n* = 120)	(*n* = 436)		
Basic Characteristics					
Age					
8 (6.9)	13 (6.5)	10 (8.3)	31	0.073	
52 (44.8)	61 (30.5)	36 (30.0)	149		
56 (48.3)	126 (63.0)	74 (61.7)	256		
Sex					
74 (63.8)	139 (69.5)	94 (78.3)	307	<0.05*	
42 (36.2)	61 (30.5)	26 (21.7)	129		
Histological classification					
15 (12.9)	43 (21.5)	38 (31.7)	96	<0.001**	
56 (48.3)	98 (49.0)	66 (55.0)	220		
40 (34.5)	47 (23.5)	9 (7.5)	96		
5 (4.3)	12 (6.0)	7 (5.8)	24		
Stages					
9 (7.8)	9 (4.5)	4 (3.3)	22	<0.05*	
15 (12.9)	20 (10.0)	3 (2.5)	38		
23 (19.8)	43 (21.5)	23 (19.2)	89		
61 (52.6)	115 (57.5)	86 (71.7)	262		
8 (6.9)	13 (6.5)	4 (3.3)	25		
Smoke status					
63 (54.3)	86 (43.0)	42 (35.0)	191	<0.05*	
53 (45.7)	114 (57.0)	78 (65.0)	245		
Metastasis					
Brain					
102 (87.9)	183 (91.5)	93 (77.5)	378	<0.01**	
14 (12.1)	17 (8.5)	27 (22.5)	58		
Bone					
101 (87.1)	160 (80.0)	79 (65.8)	340	<0.001***	
15 (12.9)	40 (20.0)	41 (34.2)	96		
Liver					
110 (94.8)	177 (88.5)	96 (80.0)	383	<0.01**	
6 (5.2)	23 (11.5)	24 (20.0)	53		
Adrenal gland					
107 (92.2)	192 (96.0)	110 (91.7)	409	0.213	
9 (7.8)	8 (4.0)	10 (8.3)	27		
Lymph node					
58 (50.0)	68 (34.0)	41 (34.2)	167	<0.01**	
58 (50.0)	132 (66.0)	79 (65.8)	269		
Intrapulmonary					
105 (90.5)	169 (84.5)	101 (84.2)	375	0.262	
11 (9.5)	31 (15.5)	19 (15.8)	61		
Pleural					
107 (92.2)	168 (81.5)	95 (79.2)	365	<0.05*	
9 (7.8)	37 (18.5)	25 (20.8)	71		
Mediastinal					
114 (98.3)	192 (96.0)	112 (93.3)	418	0.161	
2 (1.7)	8 (4.0)	8 (6.7)	18		
Peritoneum					
111 (95.7)	189 (94.5)	99 (82.5)	399	<0.01**	
5 (4.3)	11 (5.5)	21 (17.5)	37		

*p < 0.05, **p < 0.001, #Un., unknown

Among all three biomarkers, levels of CYFRA21-1significantly correlated with occurrence of organ metastasis. Besides metastasis to bone (TA: negative9.6%, moderate 25.3%, high 27.6%, *P* < 0.01; VA: negative 12.9%, moderate 20.0%, high 34.2%; *P* < 0.001) and liver (TA: negative 1.7%, moderate10.5%, high 15.6%, *P* < 0.01; VA: negative 5.2%, moderate11.5%, high 20.0%; *P* < 0.001), CYFRAY21-1 levels were also associated with metastases to lymph nodes (TA: negative 42.6%, moderate 64.2%, high 70.9%, *P* < 0.001; VA: negative 50%, moderate 66%, high 65.8%; *P* < 0.01), pleura (TA: *P* < 0.01, VA: *P* < 0.05) and peritoneum (TA: *P* < 0.01, VA: *P* < 0.01) (Table 1). However, CEA and NSE levels showed relative poor correlation with metastases (Additional file 1: Table S1A and B), which confirmed the importance of CYFRA21-1 in metastasis. Consistent results between training and validation groups also indicated the grouping rationality although several deviations such as sex, brain metastasis and adrenal gland metastasis in CYFRA21-1 and NSE, while brain and liver metastasis in CEA (Table 1, Additional file 1: Table S1A and B).

### Correlation of CYFRA21-1 and NSE with metastases in ADC and SCC, respectively

In this study, the CYFRA21-1 levels showed a stronger correlation with occurrence of metastasis in ADC patients when compared with that of CEA and NSE. It also showed a significant correlation with presence of metastatic lesions in brain (*P* < 0.05), bone (*P* < 0.001), liver (*P* < 0.05), lymph node (*P* < 0.001), intrapulmonary (*P* < 0.05), pleural (*P* < 0.05) and peritoneum (*P* < 0.05) (Table 2). However, CEA positive levels correlated only with bone (*P* < 0.05) and liver metastasis (*P* < 0.05) (Additional file 2: Table S2A), while NSE levels correlated only with metastatic lesions in brain (*P* < 0.001) and bone (*P* < 0.001) (Additional file 2: Table S2B).

**Table 2:** The association analysis between CYFRA21-1 and ADC

	No. (%)				
	Neg	Moderate	High	Total	*P* Value
		(1–3 fold)	>3 fold		
	(*n* = 120)	(*n* = 201)	(*n* = 124)	(*n* = 445)	
Basic Characteristics					
Age					
< 45 years	5 (4.2)	22 (11.0)	14 (11.3)	41	<0.05*
45–60 years	57 (47.5)	66 (32.8)	38 (30.6)	161	
> 60 years	58 (48.3)	113 (56.2)	72 (58.1)	243	
Sex					
Male	65 (54.2)	112 (55.7)	71 (57.3)	248	0.889
Female	55 (45.8)	89 (44.3)	53 (42.7)	197	
Stages					
I + II	24 (20.0)	14 (7.0)	5 (4.0)	43	<0.001**
III + IV	91 (75.8)	181 (90.0)	116 (93.6)	388	
Unknown	4 (4.2)	6 (3.0)	3 (2.4)	14	
Smoke status					
No	79 (65.9)	119 (59.2)	68 (54.8)	266	0.208
Yes	41 (34.1)	82 (40.8)	56 (45.2)	179	
Metastasis					
Brain					
No	107 (89.2)	164 (81.6)	95 (76.6)	366	<0.05*
Yes	13 (10.8)	37 (18.4)	29 (23.4)	79	
Bone					
No	102 (85.0)	141 (70.1)	75 (60.5)	318	<0.001**
Yes	18 (15.0)	60 (29.9)	49 (39.5)	127	
Liver					
No	116 (96.7)	181 (90.1)	102 (82.3)	399	<0.05*
Yes	4 (3.3)	20 (9.9)	22 (17.7)	46	
Adrenal gland					
No	115 (95.8)	191 (95.0)	116 (93.5)	422	0.713
Yes	5 (4.2)	10 (5.0)	8 (6.5)	23	
Lymph node					
No	69 (57.5)	69 (34.3)	45 (36.3)	183	<0.001**
Yes	51 (42.5)	132 (65.7)	79 (63.7)	262	
Intrapulmonary					
No	111 (92.5)	165 (82.1)	105 (84.7)	381	<0.05*
Yes	9 (7.5)	36 (17.9)	19 (15.3)	64	
Pleural					
No	103 (85.8)	161 (80.1)	88 (71.0)	352	<0.05*
Yes	17 (14.2)	40 (19.9)	36 (29.0)	93	
Mediastinal					
No	119 (99.2)	195 (97.0)	118 (95.2)	432	0.178
Yes	1 (0.8)	6 (3.0)	6 (4.8)	13	
Peritoneum					
No	119 (99.2)	186 (92.5)	107 (86.3)	412	<0.05*
Yes	1 (0.8)	15 (7.5)	17 (13.7)	33	

*p<0.05, **p<0.001

An interesting finding which differs from those reported earlier is the significant correlation of NSE levels with occurrence of metastasis in SCC patients, as compared with that of CEA and CYFRA21-1. In the present study, NSE levels were associated with occurrence of metastases to brain (*P* < 0.05), bone (*P* < 0.05), lymph nodes (*P* < 0.05), mediastinum (*P* < 0.05) and peritoneal cavity (*P* < 0.05) (Table 3). However, CEA levels correlated only with lymph node metastasis (Additional file 3: Table S3A), while CYFRA21-1 was associated with metastasis to brain (Negative: 5.6%; moderate: 2.4%; high: 16.0%, *P* < 0.05), and lymph node (Negative: 41.7%; moderate: 60%; high: 74.5%; *P* < 0.05) (Additional file 3: Table S3B).

**Table 3:** The association analysis between NSE and SCC

	No. (%)				
	Neg	Moderate	High	Total	*P* Value
		(1–2 fold)	>2 fold		
	(*n* = 110)	(*n* = 70)	(*n* = 35)	(*n* = 215)	
Basic Characteristics					
Age					
< 45 years	3 (2.7)	2 (2.9)	0 (0.0)	5	0.622
45–60 years	40 (36.4)	23 (32.8)	9 (25.7)	72	
> 60 years	67 (60.9)	45 (64.3)	26 (74.3)	138	
Sex					
Male	101 (91.8)	61 (87.1)	30 (85.7)	192	0.463
Female	9 (8.2)	9 (12.9)	5 (14.3)	23	
Stages					
I + II	26 (23.6)	6 (8.6)	1 (2.8)	33	<0.05*
III + IV	80 (72.7)	62 (88.6)	33 (94.4)	175	
Unknown	4 (3.7)	2 (2.8)	1 (2.8)	7	
Smoke status					
No	22 (20.0)	16 (22.9)	9 (25.7)	47	0.753
Yes	88 (80.0)	54 (77.1)	26 (74.3)	168	
Metastasis					
Brain					
No	107 (97.3)	62 (88.6)	27 (77.1)	196	<0.05*
Yes	3 (2.7)	8 (11.4)	8 (22.9)	19	
Bone					
No	100 (90.9)	55 (78.6)	27 (77.1)	182	<0.05*
Yes	10 (9.1)	15 (21.4)	8 (22.9)	33	
Liver					
No	102 (92.7)	61 (87.1)	27 (77.1)	190	0.062
Yes	8 (7.3)	9 (12.9)	8 (22.9)	25	
Adrenal gland					
No	106 (96.4)	64 (91.4)	32 (91.4)	202	0.316
Yes	4 (3.6)	6 (8.6)	3 (8.6)	13	
Lymph node					
No	51 (46.4)	19 (27.1)	9 (25.7)	79	<0.05*
Yes	59 (53.6)	51 (72.9)	26 (74.3)	136	
Intrapulmonary					
No	96 (87.3)	58 (82.9)	31 (88.6)	185	0.632
Yes	14 (12.7)	12 (17.1)	4 (11.4)	30	
Pleural					
No	98 (89.1)	59 (84.3)	31 (88.6)	188	0.622
Yes	12 (10.9)	11 (15.7)	4 (11.4)	27	
Mediastinal					
No	108 (98.2)	61 (87.1)	34 (97.1)	203	<0.05*
Yes	2 (1.8)	9 (12.9)	1 (2.9)	12	
Peritoneum					
No	109 (99.1)	61 (87.1)	31 (88.6)	201	<0.05*
Yes	1 (0.9)	9 (12.9)	4 (11.4)	14	

*p < 0.05, **p < 0.001

In the present study, 18.3% of all subjects were small cell lung cancer (SCLC) patients. In these patients, we observed a correlation between increased levels of CEA and occurrence of mediastinal and peritoneal metastasis (*P* < 0.05) (Additional file 4: Table S4A); between increased levels of CYFRA21-1 and liver metastasis (*P* < 0.05) (Additional file 4: Table S4B); and between increased NSE levels and occurrence of lymph node metastasis (Negative: 42.1%; moderate: 60.1%; high: 77.8%;*P* < 0.05) (Additional file 4: Table S4C).

### Increased positive numbers of biomarkers as predictors of metastases

The analysis of increased positive numbers of biomarkers in all lung cancer patients was performed in training group and validation groups. In training group, the numbers of negative, single, double and triple groups were 37, 101, 172 and 122 cases, respectively, while 27, 118, 161 and 130 in the validation group. The number TA and VA groups indicated less data deviation among different groups. The results suggested strong correlation of increased positive numbers with stages (TA: *P* < 0.05, VA: *P* < 0.05). In metastasis analysis, increased positive numbers related closely to occurrence of metastasis in bone (TA: Neg 10.8%, Single 13.9%, Double 26.2%, Triple 27.9%, *P* < 0.05; VA: Neg 0%, Single 12.7%, Double 23.6%, Triple 33.1%, *P* < 0.001) and lymph node (TA: Neg 32.4%, Single 55.4%, Double 59.9%, Triple 73.8%, *P* < 0.001; VA: Neg 29.6%, Single 50.8%, Double 68.9%, Triple 69.2%, *P* < 0.001) (Table 4).

**Table 4:** The analysis of positive numbers of biomarkers in all lung cancer patients

	No. (%)					
	Neg	Single	Double	Triple	Total	*P* Value
		(1–10 fold)	>10 fold			
	(*n* = 37)	(*n* = 101)	(*n* = 172)	(*n* = 122)	(*n* = 432)	
Basic Characteristics						
Age						
< 45	2 (5.4)	5 (5.0)	12 (7.0)	9 (7.4)	28	0.057
45-60	22 (59.5)	50 (49.5)	61 (35.5)	43 (35.2)	176	
> 60	13 (35.1)	46 (45.5)	99 (57.6)	70 (57.4)	228	
Sex						
Male	27 (73.0)	71 (70.3)	114 (66.3)	84 (68.9)	296	0.827
Female	10 (27.0)	30 (29.7)	58 (33.7)	38 (31.1)	136	
Histological classification						
SCC	12 (32.4)	29 (28.7)	49 (28.5)	29 (23.8)	119	0.772
ADC	19 (51.4)	53 (52.5)	90 (52.3)	63 (51.6)	225	
SCLC	3 (8.1)	12 (11.9)	24 (14)	24 (19.7)	63	
Others	3 (8.1)	7 (6.9)	9 (5.2)	6 (4.9)	25	
Stages						
I	5 (13.5)	8 (7.9)	5 (2.9)	0 (0)	18	<0.05*
II	2 (5.4)	6 (5.9)	5 (2.9)	4 (3.3)	17	
III	14 (37.8)	34 (33.7)	43 (25)	37 (30.3)	128	
IV	14 (37.8)	47 (46.5)	109 (63.4)	73 (59.8)	243	
#Un.	2 (5.4)	6 (5.9)	10 (5.8)	8 (6.6)	26	
Smoke status						
No	16 (43.2)	54 (53.5)	73 (42.4)	48 (39.3)	191	0.178
Yes	21 (56.8)	47 (46.5)	99 (57.6)	74 (60.7)	241	
Metastasis						
Brain						
No	34 (91.9)	91 (90.1)	143 (83.1)	103 (84.4)	371	0.277
Yes	3 (8.1)	10 (9.9)	29 (16.9)	19 (15.6)	61	
Bone						
No	33 (89.2)	87 (86.1)	127 (73.8)	88 (72.1)	335	<0.05*
Yes	4 (10.8)	14 (13.9)	45 (26.2)	34 (27.9)	97	
Liver						
No	36 (97.3)	94 (93.1)	155 (90.1)	106 (86.9)	391	0.199
Yes	1 (2.7)	7 (6.9)	17 (9.9)	16 (13.1)	41	
Adrenal gland						
No	36 (97.3)	98 (97)	154 (89.5)	117 (95.9)	405	0.086
Yes	1 (1.7)	3 (3.0)	18 (10.5)	5 (4.1)	27	
Lymph node						
No	25 (67.6)	45 (44.6)	69 (40.1)	32 (26.2)	171	<0.001**
Yes	12 (32.4)	56 (55.4)	103 (59.9)	90 (73.8)	261	
Intrapulmonary						
No	33 (89.2)	89 (88.1)	149 (86.6)	108 (88.5)	379	0.950
Yes	4 (10.8)	12 (11.9)	23 (13.4)	14 (11.5)	53	
Pleural						
No	33 (89.2)	89 (88.1)	142 (82.6)	104 (85.2)	368	0.552
Yes	4 (10.8)	12 (11.9)	30 (17.4)	18 (14.8)	64	
Mediastinal						
No	37 (100)	98 (97)	169 (98.3)	114 (93.4)	418	0.080
Yes	0 (0.0)	3 (3.0)	3 (1.7)	8 (6.6)	14	
Peritoneum						
No	37 (100)	98 (93.7)	162 (94.2)	112 (91.8)	409	0.153
Yes	0 (0.0)	3 (6.3)	10 (5.8)	10 (8.2)	23	
Validation group						
	No. (%)					
	Neg	Single	Double	Triple	Total	*P* Value
	(*n* = 27)	(*n* = 118)	(*n* = 161)	(*n* = 130)	(*n* = 436)	
Basic Characteristics						
Age						
2 (7.4)	8 (6.8)	10 (6.2)	11 (8.5)	31	0.733	
10 (37.0)	46 (39.0)	48 (29.8)	45 (34.6)	149		
15 (55.6)	64 (54.2)	103 (64.0)	74 (56.9)	256		
Sex						
18 (66.7)	75 (63.6)	116 (72.0)	98 (75.4)	307	0.204	
9 (33.3)	43 (36.4)	45 (28.0)	32 (24.6)	129		
Histological classification						
7 (25.9)	31 (26.3)	37 (23)	21 (16.2)	96	0.386	
15 (55.6)	57 (48.3)	84 (52.2)	64 (49.2)	220		
1 (3.7)	5 (4.2)	11 (6.8)	7 (5.4)	24		
5 (18.5)	12 (10.2)	7 (4.3)	24 (18.5)	47		
Stages						
5 (18.5)	9 (7.6)	5 (3.1)	3 (2.3)	22	<0.05*	
5 (18.5)	16 (13.6)	10 (6.2)	7 (5.4)	38		
6 (22.2)	26 (22.0)	36 (22.4)	21 (16.2)	89		
11 (40.7)	58 (49.2)	103 (64.0)	90 (69.2)	262		
0 (0.0)	9 (7.6)	7 (4.3)	9 (19.2)	25		
Smoke status						
15 (55.6)	62 (52.5)	65 (40.4)	49 (37.7)	191	<0.05*	
12 (44.4)	56 (47.5)	96 (59.6)	81 (62.3)	245		
Metastasis						
Brain						
27 (100.0)	107 (90.7)	134 (83.2)	110 (84.6)	378	<0.05*	
0 (0.0)	11 (9.3)	27 (16.8)	20 (15.4)	58		
Bone						
27 (100.0)	103 (87.3)	123 (76.4)	87 (66.9)	340	<0.001**	
0 (0.0)	15 (12.7)	38 (23.6)	43 (33.1)	96		
Liver						
No	26 (96.3)	111 (94.1)	140 (87.0)	106 (81.5)	383	<0.05*
Yes	1 (3.7)	7 (5.9)	21 (13.0)	24 (18.5)	53	
Adrenal gland						
27 (100.0)	111 (94.1)	149 (92.5)	122 (93.8)	409	0.525	
0 (0.0)	7 (5.9)	12 (7.5)	8 (6.2)	27		
Lymph node						
19 (70.4)	58 (49.2)	50 (31.1)	40 (30.8)	167	<0.001***	
8 (29.6)	60 (50.8)	111 (68.9)	90 (69.2)	269		
Intrapulmonary						
26 (96.3)	105 (89.0)	130 (80.7)	114 (87.7)	375	0.064	
1 (3.7)	13 (11.0)	31 (19.3)	16 (12.3)	61		
Pleural						
25 (92.6)	107 (90.7)	129 (80.1)	104 (80.0)	365	<0.05*	
2 (7.4)	11 (9.3)	32 (20.8)	26 (20.0)	71		
Mediastinal						
27 (100.0)	116 (98.3)	152 (94.4)	123 (94.6)	418	0.229	
0 (0.0)	2 (1.7)	9 (5.6)	7 (5.4)	18		
Peritoneum						
27 (100.0)	110 (93.2)	144 (89.4)	118 (90.8)	399	0.269	
0 (0.0)	8 (6.8)	17 (10.4)	12 (9.2)	37		

The application of 3-tier classification to all types of lung cancers revealed that lymph node metastasis was significantly associated with increased levels of biomarkers (ADC *P* < 0.05; SCC *P* < 0.001; SCLC *P* < 0.05) (Additional file 5: Table S5A-C). In ADC and SCC, increased levels correlated with metastasis to both lymph nodes and other organs (Additional file 5: Table S5A-C).

### CYFRA21-1 levels correlated with survival in ADC, SCC and SCLC

**Fig. 1:**
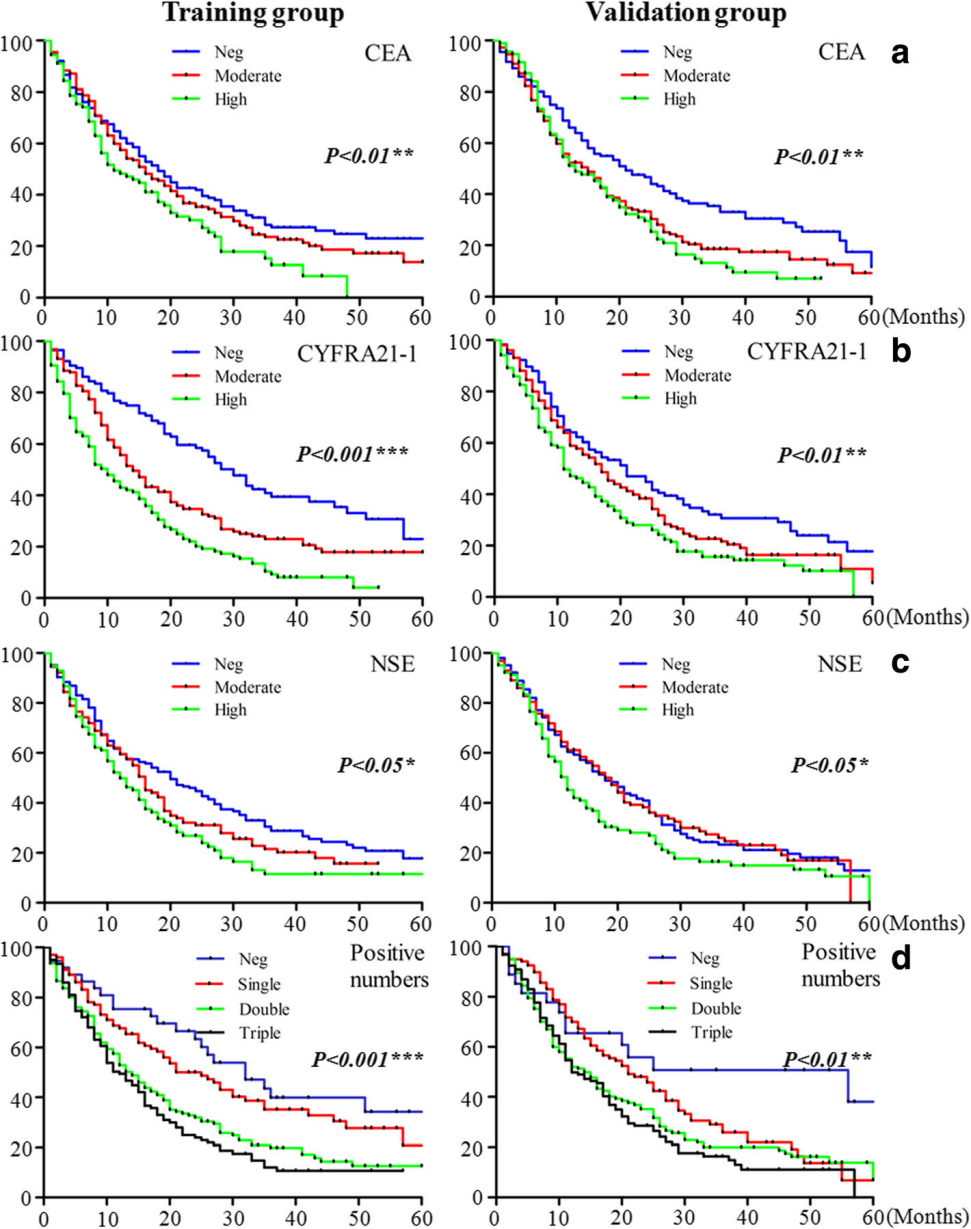
The survival status of lung cancer patients in training and validation groups **a**: CEA, **b**: CYFRA21-1, **c**: NSE, **d**: positive numbers *P < 0.05, **P < 0.001

Kaplan-Meier survival curves were used to analyze mortality at 3–5 years using SPSS19.0. The results of 3–5 year survival analyses indicated that presence of high concentrations of CEA (TA *P* < 0.01; VA *P* < 0.01), CYFRA21-1 (TA *P* < 0.001; VA *P* < 0.001), NSE (TA *P* < 0.05; VA *P* < 0.05) and positive numbers of biomarkers (TA *P* < 0.001; VA *P* < 0.01) were closely associated with survival status in both training group and validation groups (Fig. 1a-d).

**Fig. 2:**
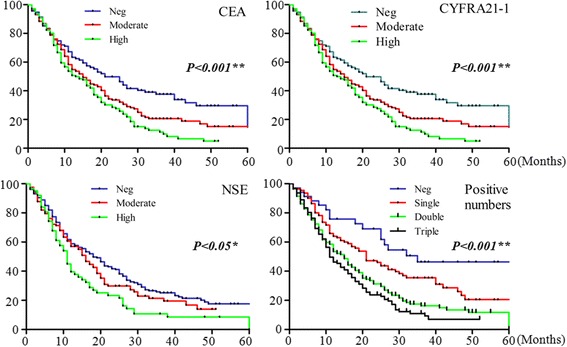
The survival functions in ADC patients correlated with different biomarkers *P < 0.05, **P < 0.001

For ADC patients, high levels of CEA (*P* < 0.001), CYFRA21-1 (*P* < 0.001), NSE (*P* < 0.05), and numbers of increased biomarkers (*P* < 0.001), were all closely associated with survival status of patients (Fig. 2). In SCC patients only CYFRA21-1 was associated with mortality (Additional file 6: Figure S1A). In SCLC patients, the high concentrations of CYFRA21-1 (*P* < .05) and NSE (*P* < .05) were closely associated with survival status (Additional file 7: Figure S1B).

### Multivariate Cox regression analysis to identify poor prognostic factors

We observed a significant correlation between overall survival and CYFRA21-1, NSE and occurrence of metastasis. Compared with negative group, the hazards ratio increased 1.226 in CYFRA21-1 moderate positive group (Confidence Interval [CI]: 0.977–1.537) and 1.647 in CYFRA21-1 high positive group (CI: 1.273–2.130, *P* < .001) (Table 5). For NSE, we did not find a significant difference between hazard risk and NSE moderate positive group (HR: 1.010, CI: 0.808–1.263) but the HR increased 1.291 in NSE high positive group compared with that of negative group (CI: 1.032–1.715, *P* < .05). As expected, occurrence of metastasis was an independent predictor of poor prognosis (HR: 1.291, CI: 1.025–1.625, *P* < .05) (Table 5).

**Table 5:** The multivariate analysis of lung cancer patients

	Multivariate HR (95% CI)	*P* value
Age		
< 45	1[Reference]	<0.001*
45–65	0.714 (0.513–0.994)	
> 65	1.089 (0.793–1.495)	
Sex		
Male	1[Reference]	0.529
Female	0.942 (0.782–1.135)	
Histological classification		
Squamous	1[Reference]	<0.05*
Adenocarcinoma	1.113 (0.894–1.384)	
SCLC	0.970 (0.729–1.290)	
Others	1.654 (1.160–2.358)	
Stages		
I	1[Reference]	<0.05*
II	1.096 (0.624–1.925)	
III	1.218 (0.753–1.969)	
IV	1.976 (1.120–3.488)	
Smoke statues		
No	1[Reference]	0.095
Yes	0.823 (0.655–1.035)	
CEA levels		
Neg	1[Reference]	0.233
Moderate	1.171 (0.954–1.438)	
High	1.217 (0.945–1.567)	
CYFRA levels		
Neg	1[Reference]	<0.001*
Moderate	1.226 (0.977–1.537)	
High	1.647 (1.273–2.130)	
NSE levels		
Neg	1[Reference]	<0.05*
Moderate	1.010 (0.808–1.263)	
High	1.330 (1.032–1.715)	
Metastasis		
No	1[Reference]	<0.05*
Yes	1.291 (1.025–1.625	
Positive numbers		
Neg	1[Reference]	0.649
Single	1.075 (0.806–1.434)	
Double	1.102 (0.898–1.353)	
Triple	1.086 (0.773–1.524)	

The specific histological grade analysis indicated that high and moderate levels of serum CYFRA21-1 significantly correlated with poor prognosis (HR: 1.860, CI: 1.036–3.338, *P* < 0.05) in both ADC and SCLC patients (HR: 1.365, CI: 0.514–3.624, *P* < 0.05) respectively (Table 6). In SCC and SCLC patients, only occurrence of metastasis was an independent factor for poor prognosis (Table 6). When compared with negative groups, the number of positive biomarkers meant increased mortality risk in SCLC (Single: HR 2.107, CI 0.460–9.644; double: HR 2.247 CI 0.386–13.077; triple: HR 2.508, CI 0.231–27.287) (Table 6) although the associated *P* value was >0.05.

**Table 6:** The multivariate analysis of different histological classifications

	Adenocarcinoma		Squamous		SCLC	
	(*n* = 445)		(*n* = 215)		(*n* = 159)	
	HR (95% CI)	*P* value	HR (95% CI)	*P* value	HR (95% CI)	*P* value
Age						
< 45	1[Reference]	<0.05*	1[Reference]	<0.05*	1[Reference]	0.104
45–65	0.733 (0.489–1.099)		0.866 (0.259–2.895)		0.769 (0.315–1.876)	
> 65	1.084 (0.741–1.587)		1.712 (0.523–5.607)		1.237 (0.510–3.003)	
Sex						
Male	1[Reference]	0.338	1[Reference]	0.326	1[Reference]	0.354
Female	0.986 (0.715–1.122)		1.312 (0.763–2.254)		0.758 (0.421–1.363)	
Stages						
I + II	1[Reference]	0.415	1[Reference]	0.475	1[Reference]	0.902
III + IV	1.703 (1.035–2.802)		0.832 (0.495–1.399)		1.091 (0.465–2.556)	
Smoke status						
No	1[Reference]	0.177	1[Reference]	0.878	1[Reference]	0.076
Yes	0.807 (0.592–1.102)		1.037 (0.651–1.651)		0.518 (0.251–1.071)	
CEA levels						
Neg	1[Reference]	0.773	1[Reference]	0.295	1[Reference]	0.940
Moderate	1.085 (0.679–1.736)		1.244 (0.620–2.497)		0.850 (0.317–2.280)	
High	1.169 (0.713–1.916)		0.700 (0.260–1.885)		0.894 (0.271–2.942)	
CYFRA levels						
Neg	1[Reference]	<0.05*	1[Reference]	0.195	1[Reference]	<0.05*
Moderate	1.161 (0.678–1.989)		1.057 (0.511–2.185)		1.365 (0.514–3.624)	
High	1.860 (1.036–3.338)		1.502 (0.673–3.353)		0.907 (0.285–2.880)	
NSE levels						
Neg	1[Reference]	0.400	1[Reference]	0.329	1[Reference]	0.642
Moderate	1.025 (0.727–1.446)		1.025 (0.727–1.446)		0.952 (0.390–2.323)	
High	1.154 (0.777–1.714)		1.154 (0.777–1.714)		1.342 (0.590–3.052)	
Metastasis						
No	1[Reference]	0.477	1[Reference]	<0.05*	1[Reference]	<0.05*
Yes	1.131 (0.806–1.585)		1.682 (1.052–2.688)		2.172 (1.180–3.998)	
Positive numbers						
Neg	1[Reference]	0.852	1[Reference]	0.334	1[Reference]	0.814
Single	1.334 (0.672–2.649)		0.748 (0.300–1.863)		2.107 (0.460–9.644)	
Double	1.491 (0.557–3.992)		1.115 (0.327–3.803)		2.247 (0.386–13.077)	
Triple	1.652 (0.517–5.276)		0.901 (0.183–4.449)		2.508 (0.231–27.287)	

Lung cancer patients were then divided into three groups according to stages (I + II, III and IV) and Cox regression was conducted to analyze which biomarker could act as independent factor of poor prognosis in specific stage. The results indicated that CYFRA21-1 correlated dramatically with poor prognosis in all stages of lung cancer patients (Stages I-II: HR 3.666 CI: 1.095–12.279, *P* < 0.05; Stage III: HR 1.919 CI: 1.200–3.071, *P* < 0.05; Stage IV: HR 1.473 CI: 1.056–2.053, *P* < 0.05) (Table 7 A-C), which confirm the importance of CYFRA21-1 in poor prognosis in different stages of lung cancer besides in specific histological classifications.

**Table 7:** Cox regression analysis of CEA, CYFRA21-1 and NSE in different stages of lung cancer

	Multivariate HR (95% CI)	*P* value
A I + II		
Age		
< 45	1[Reference]	0.405
45–65	0.390 (0.043–3.577)	
> 65	0.664 (0.075–5.874)	
Sex		
Male 1[Reference]		0.997
Female	0.998 (0.358–2.779)	
Smokes		
No	1[Reference]	0.828
Yes	1.091 (0.496–2.400)	
Histological classification		
SCC	1[Reference]	0.400
ADC	0.692 (0.294–1.631)	
SCLC	1.000 (0.347–2.884)	
Unknown	0.943 (0.242–3.670)	
Metastasis		
No	1[Reference]	0.992
Yes	0.997 (0.505–1.967)	
CEA		
Neg	1[Reference]	0.483
Moderate	1.213 (0.555–2.651)	
High	1.442 (0.519–4.009)	
NSE		
Neg	1[Reference]	0.592
Moderate	1.064 (0.542–2.090)	
High	0.718 (0.214–2.411)	
CYFRA		
Neg	1[Reference]	<0.05*
Moderate	1.696 (0.848–3.390)	
High	3.666 (1.095–12.279)	
B. Stage III		
Age		
< 45	1[Reference]	0.147
45–65	0.492 (0.189–1.283)	
> 65	1.230 (0.491–3.083)	
Sex		
Male	1[Reference]	0.934
Female	0.976 (0.555–1.718)	
Smokes		
No	1[Reference]	0.758
Yes	1.075 (0.680–1.699)	
Histological classification		
SCC	1[Reference]	0.272
ADC	0.974 (0.624–1.521)	
SCLC	0.796 (0.445–1.424)	
Unknown	1.439 (0.752–2.756)	
Metastasis		
No	1[Reference]	0.094
Yes	1.444 (0.939–0.221)	
CEA		
Neg	1[Reference]	0.423
Moderate	1.047 (0.715–1.532)	
High	1.260 (0.716–2.218)	
NSE		
Neg	1[Reference]	0.165
Moderate	0.738 (0.480–1.134)	
High	1.333 (0.796–2.323)	
CYFRA		
Neg	1[Reference]	<0.05*
Moderate	1.279 (0.844–1.938)	
High	1.919 (1.200–3.071)	
C Stage IV		
Age		
< 45	1[Reference]	0.285
45–65	0.818 (0.566–1.182)	
> 65	1.052 (0.739–1.499)	
Sex		
Male	1[Reference]	0.452
Female	1.125 (0.827–1.531)	
Smokes		
No	1[Reference]	0.130
Yes	1.261 (0.934–1.702)	
Histological classification		
SCC	1[Reference]	0.090
ADC	1.299 (0.960–1.756)	
SCLC	1.182 (0.801–1.744)	
Unknown	1.811 (1.082–3.030)	
Metastasis		
No	1[Reference]	<0.05*
Yes	1.494 (1.034–2.160)	
CEA		
Neg	1[Reference]	0.332
Moderate	1.132 (0.881–1.456)	
High	1.074 (0.802–1.439)	
NSE		
Neg	1[Reference]	0.060
Moderate	1.042 (0.806–1.346)	
High	1.319 (0.989–1.759)	
CYFRA		
Neg	1[Reference]	<0.05*
Moderate	1.107 (0.822–1.489)	
High	1.473 (1.056–2.053)	

## Discussion

Although several tumor markers for lung cancer have been identified, none of them is specific for lung cancer diagnosis. CYFRA21-1 has been reported as a poor prognostic factor in various cancers, while NSE has been associated with metastasis, and also used for monitoring response to treatment in multiple myeloma. However, these important biomarkers have not been thoroughly investigated in lung cancer patients. In our study, analyses were performed to confirm the correlations between serums CEA, CYFRA 21–1, NSE, as well as the number of positive biomarkers and metastasis and survival status of lung cancer patients.

All patients were randomly divided into training and validation groups to confirm the grouping rationality of this study. In brief, survival curves and associations with clinical characteristics in the validation group were generally similar to those in training group, though there were some non-significant inconsistence in sex and several organs of metastasis. The results indicated that the increased levels of CYFRA21-1 were strongly associated with metastatic sites and histological grades of lung cancers in both training and validation groups. In specific histological subtypes (ADC, SCC and SCLC) analyses, we also found that CYFRA21-1 correlated more closely to metastasis and survival status than CEA and NSE. To our knowledge, these results have not been reported in any of the earlier literatures.

In multivariate Cox regression analysis, only CYFRA21-1 and NSE were found to be independent predictors of prognosis in lung cancer patients. When sub-grouped, only CYFRA21-1was an independent predictor of poor prognosis in ADC (1.86-fold increased risk in high concentration group) and SCLC (1.365-fold increased risk in moderate group) but not CEA and NSE. Finally it was found that CYFRA21-1 could act as independent factor in early (I + II) and advanced stages (III and IV) of lung cancer. Thus, CYFRA21-1 appears to be more important as a prognostic predictor than the other two biomarkers.

Several reports have reported the useful roles of CEA in diagnosis of ADC, CYFRA21-1 in SCC and NSE in SCLC [18–21]. The increased levels of biomarkers are known to correlate with cancer progression, with their sensitivity depending largely on tumor stage and histological classification [22]. In contrast with the previous reports [25], we found no correlation between increased CEA levels and brain metastasis; however, we did observe a correlation with bone, liver, pleural and peritoneal metastases. The inconsistency could be explained by the smaller sample size (approximate *N* = 300). Research also indicated that high circulating concentrations of CYFRA21-1 and CEA were associated with advanced stages of lung cancer; levels that were two times higher than cutoff value were associated with stage III and IV lung cancer patients [23]. Although CYFRA21-1 appears to be the most sensitive and specific marker for NSCLC [26], its relationship with different histological lung cancers has largely remained unknown. An earlier report suggested that CYFRA was a more sensitive and specific marker for SCC diagnosis and was found to be of prognostic values in patients with recurrent NSCLC receiving gefitinib therapy [27, 28]. In our study, however, high levels of CYFRA21-1 correlated with survival status in ADC and SCLC but not in SCC patients. It also could be used as an independent predictor of poor prognosis in ADC and SCLC patients. Currently, NSE is the most widely used marker for diagnosis and prognosis of SCLC patients [24]. In our study, although positive levels of NSE significantly correlated with survival in SCLC, it did not qualify as an independent predictor for poor prognosis.

## Conclusions

We designed this study to evaluate whether positive levels of biomarkers correlate with occurrence of metastasis and poor survival. The retrospective design and cross-sectional nature of our study are limitations that did not permit correlation analysis for all clinic pathological parameters. Our study suggested the important role of CYFRA21-1 in predicting occurrence of metastasis as well as poor prognosis in lung cancer patients. Our results could provide important perspectives for diagnosis, prognosis and management of lung cancer.

## Additional files

Additional file 1: Table S1. Association analysis between CEA, NSE and all lung cancer patients. (DOCX 36 kb)
Additional file 2: Table S2. The association analysis between CEA, NSE positive levels and ADC patients. (DOC 55 kb)
Additional file 3: Table S3. The association analysis between bio-markers positive levels and SCC. (DOC 65 kb)
Additional file 4: Table S4. The association analysis between bio-markers positive levels and SCLC. (DOC 63 kb)
Additional file 5: Table S5. The association analysis between positive numbers and ADC, SCC, SCLC patients. (DOC 59 kb)
Additional file 6: Figure S1A. Survival functions between SCC patients and the level of CEA, CYFRA21-1, NSE and positive numbers. **P* < 0.05, ***P* < 0.001. (TIF 1909 kb)
Additional file 7: Figure S1B. The survival functions analysis in SCLC patients based on increased concentrations in CEA, CYFRA21-1, NSE and positive numbers of biomarkers. **P* < 0.05, ***P* < 0.001. (TIF 1899 kb)

## Abbreviations

ADC: Adenocarcinoma
CEA: Carcinoembryonic antigen
CT: Computed tomography
CYFRA21-1: Cytokeratin 19 fragments
EBC: Exhaled breath condensate
HR: Hazard ration
NSCLC: Non-small cell lung cancer
NSE: Neuron-specific enolase
SCC: Squamous cell carcinoma
SCLC: Small cell lung cancer
TA: Training
VA: Validation
