# Supplementary material for: The important role of circulating CYFRA21-1 in metastasis diagnosis and prognostic value compared with carcinoembryonic antigen and neuron-specific enolase in lung cancer patients
Source: BMC Cancer. 2017 Feb 2;17:96. doi: 10.1186/s12885-017-3070-6 (PMC5290605; doi:10.1186/s12885-017-3070-6)
Supplement: Additional file 1: Table S1. — Association analysis between CEA, NSE and all lung cancer patients. (DOCX 36 kb) [file 12885_2017_3070_MOESM1_ESM.docx]

Additional file 1: Table S1. Association analysis between CEA, NSE and all lung cancer patients

**A CEA**

**Validation group**

**Training group**

No. (%)

No. (%)

Neg Moderate High Total P Value Neg Moderate High Total P Value

1-10 fold >10 fold 1-10 fold >10 fold

(n=164) (n=179) (n=89) (n=432) (n=156) (n=186) (n=94) (n=436)

**Basic Characteristics Basic Characteristics**

Age Age

<45 9(5.5) 14(7.8) 5(5.6) 28 8(5.1) 16(8.6) 7(7.4) 31

0.631

0.199

45-60 73(44.5) 61(34.1) 42(47.2) 176 53(34.0) 60(32.3) 36(38.3) 149

>60 82(50.0) 104(58.1) 42(47.2) 228 95(60.9) 110(59.1) 51(54.3) 256

Sex Sex

Male 119(72.6) 128(71.5) 49(55.1) 296 112(71.8) 135(72.6) 60(63.8) 307

***<0.01*****

0.284

Female 45(27.4) 51(28.5) 40(44.9) 136 44(28.2) 51(27.4) 34(36.2) 129

Histological classification Histological classification

SCC 64(39.0) 48(26.8) 7(7.9) 119 53(34.0) 38(20.4) 5(5.3) 96

ADC 61(37.2) 95(53.1) 69(77.5) 225 54(34.6) 94(50.5) 72(76.5) 220

***<0.001******

***<0.001******

SCLC 27(16.5) 28(15.6) 8(9.0) 63 40(25.6) 46(24.8) 10(10.6) 96

Others 12(7.3) 8(4.5) 5(5.6) 25 9(5.8) 8(4.3) 7(7.5) 24

Stages Stages

I 13(7.9) 4(2.2) 1(1.1) 18 14(9.0) 5(2.7) 3(3.2) 22

II 6(3.7) 9(5.0) 2(2.2) 17 20(12.8) 14(7.5) 4(4.3) 38

***<0.001******

***<0.01*****

III 56(34.1) 54(30.2) 18(20.2) 128 38(24.4) 36(19.4) 15(16.0) 89

IV 79(48.2) 103(57.5) 61(68.5) 243 73(46.8) 121(65.0) 68(72.3) 262

^#^Un. 10(6.1) 9(5.1) 7(8.0) 26 11(7.0) 10(5.4) 4(4.2) 25

Smoke status Smoke status

No 69(42.1) 74(41.3) 48(53.9) 191 67(42.9) 78(41.9) 46(48.9) 191

0.518

0.116

Yes 95(57.9) 105(58.7) 41(46.1) 241 89(57.1) 108(58.1) 48(51.1) 245

**Metastasis Metastasis**

Brain Brain

***<0.05****

No 142(86.6) 155(86.6) 74(83.1) 371 143(91.7) 159(85.3) 76(80.9) 378

0.695

Yes 22(13.4) 24(13.4) 15(16.9) 61 13(8.3) 27(14.5) 18(19.1) 58

Bone Bone

No 134(81.7) 139(77.7) 60(67.4) 335 134(85.9) 148(79.6) 58(61.7) 340

***<0.01*****

***<0.05****

Yes 30(18.3) 40(22.3) 29(32.6) 97 22(14.1) 38(20.4) 36(38.3) 96

Liver Liver

No 145(88.4) 169(94.4) 77(86.5) 391 143(91.7) 164(88.2) 76(80.9) 383

***<0.05****

0.060

Yes 19(11.6) 10(5.6) 12(13.5) 41 13(8.3) 22(11.8) 18(19.1) 53

Adrenal gland Adrenal gland

No 153(93.3) 170(95.0) 82(92.1) 405 145(92.9) 176(94.6) 88(93.6) 409

0.634

0.213

Yes 11(6.7) 9(5.0) 7(7.9) 27 11(7.1) 10(5.4) 6(6.4) 27

Lymph node Lymph node

No 69(42.1) 69(38.5) 33(37.1) 171 71(45.5) 68(33.9) 33(35.1) 167

0.068

0.691

Yes 95(57.9) 110(61.5) 56(62.9) 261 85(54.5) 123(66.1) 61(64.9) 269

Intrapulmonary Intrapulmonary

No 143(87.2) 162(90.5) 74(83.1) 379 135(86.5) 163(87.6) 77(81.9) 375

0.217

0.416

Yes 21(12.8) 17(9.5) 15(16.9) 53 21(13.5) 23(12.4) 17(18.1) 61

Pleural Pleural

No 143(87.2) 153(85.5) 72(80.9) 368 137(87.8) 156(83.9) 72(76.6) 365

0.066

0.400

Yes 21(12.8) 26(14.5) 17(19.1) 64 19(12.2) 30(16.1) 22(23.4) 71

Mediastinal Mediastinal

No 159(97.0) 174(97.2) 85(95.5) 418 151(96.8) 181(97.3) 86(91.5) 418

0.748

0.053

Yes 5(3.0) 5(2.8) 4(4.5) 14 5(3.2) 5(2.7) 8(8.5) 18

Peritoneum Peritoneum

No 155(94.5) 171(95.5) 83(93.3) 409 142(91.0) 178(95.7) 79(84.0) 399

***<0.05****

0.105

Yes 9(5.5) 8(4.5) 6(6.7) 23 14(9.0) 8(4.3) 15(16.0) 37

*p<0.05, **p<0.001, ^#^Un., unknown

**B NSE**

**Validation group**

**Training group**

No. (%)

No. (%)

Neg Moderate High Total P Value Neg Moderate High Total P Value

1-2 fold >2 fold 1-2 fold >2 fold

(n=206) (n=128) (n=98) (n=432) (n=206) (n=128) (n=102) (n=436)

**Basic Characteristics Basic Characteristics**

Age Age

<45 14(6.8) 6(4.7) 8(8.2) 28 16(7.8) 7(5.5) 8(7.8) 31

0.771

0.103

45-60 96(46.6) 44(34.4) 36(36.7) 176 65(31.6) 48(37.5) 36(35.3) 149

>60 96(46.6) 78(60.9) 54(55.1) 228 125(60.6) 73(57.0) 58(56.9) 256

Sex Sex

Male 139(67.5) 78(60.9) 79(80.6) 296 134(65.0) 98(76.6) 75(73.5) 307

0.059

***<0.05****

Female 67(32.5) 50(39.1) 19(19.4) 136 72(35.0) 30(23.4) 27(26.5) 129

Histological classification Histological classification

SCC 58(28.2) 37(28.9) 24(24.5) 119 52(25.2) 33(25.8) 11(10.8) 96

ADC 128(62.1) 66(51.6) 31(31.6) 225 133(64.6) 56(43.8) 31(30.4) 220

***<0.001******

***<0.001******

SCLC 8(3.9) 15(11.7) 40(40.8) 63 11(5.3) 26(20.3) 59(57.8) 96

Others 12(5.8) 10(7.8) 3(3.1) 25 10(4.9) 13(10.1) 1(1.0) 24

Stages Stages

I 11(5.3) 6(4.7) 1(1.0) 18 15(7.3) 5(3.9) 2(2.0) 22

II 10(4.9) 4(3.1) 3(3.1) 17 22(10.7) 12(9.4) 4(3.9) 38

0.698

0.058

III 57(27.7) 39(30.5) 32(32.6) 128 45(21.8) 24(18.8) 20(19.6) 89

IV 117(56.8) 72(56.2) 54(55.1) 243 118(57.3) 74(57.8) 70(68.6) 262

^#^Un. 11(5.3) 7(5.5) 8(8.2) 26 6(2.9) 13(10.1) 6(5.9) 25

Smoke status Smoke status

No 105(51.0) 59(46.1) 27(27.6) 191 104(50.5) 52(40.6) 35(34.3) 191

***<0.01*****

***<0.05****

Yes 101(49.0) 69(53.9) 71(72.4) 241 102(49.5) 76(59.4) 67(65.7) 245

**Metastasis Metastasis**

Brain Brain

***<0.01*****

No 181(87.9) 107(83.6) 83(84.7) 371 184(89.3) 108(84.3) 86(84.3) 378

0.513

Yes 25(12.1) 21(16.4) 15(15.3) 61 22(10.7) 20(15.6) 16(15.7) 58

Bone Bone

No 165(80.1) 94(73.4) 76(77.6) 335 175(85.0) 91(71.1) 74(72.5) 340

***<0.01*****

***<0.001******

Yes 14(19.9) 34(26.6) 22(22.4) 97 31(15.0) 37(28.9) 28(27.5) 96

Liver Liver

No 193(93.7) 113(88.3) 85(86.7) 391 187(90.8) 116(90.6) 80(78.4) 383

***<0.001******

***<0.01*****

Yes 13(6.3) 15(11.7) 13(13.3) 41 19(9.2) 12(9.4) 22(21.6) 53

Adrenal gland Adrenal gland

No 192(93.2) 123(96.1) 90(91.8) 405 200(97.1) 119(93.0) 90(88.2) 409

***<0.05****

0.383

Yes 14(6.8) 5(3.9) 8(8.2) 27 6(2.9) 9(7.0) 12(11.8) 27

Lymph node Lymph node

No 99(48.1) 42(32.8) 30(30.6) 171 94(45.6) 49(38.3) 24(23.5) 167

***<0.01*****

***<0.01*****

Yes 107(51.9) 86(67.2) 68(69.4) 261 112(54.4) 79(61.7) 78(76.5) 269

Intrapulmonary Intrapulmonary

No 178(86.4) 114(89.1) 87(88.8) 379 178(86.4) 110(85.9) 87(85.3) 375

0.724

0.965

Yes 28(13.6) 14(10.9) 11(11.2) 53 28(13.6) 18(14.1) 15(14.7) 61

Pleural Pleural

No 176(85.4) 112(87.5) 80(81.6) 368 174(84.5) 102(79.7) 89(87.3) 365

0.464

***<0.05****

Yes 30(14.6) 16(12.5) 18(18.4) 64 32(15.5) 26(20.3) 13(12.7) 71

Mediastinal Mediastinal

***<0.01*****

No 204(99.0) 121(94.5) 93(94.9) 418 200(97.0) 121(93.8) 98(96.1) 418

***<0.05****

Yes 2(1.0) 7(5.5) 5(5.1) 14 6(3.0) 8(8.2) 4(3.9) 18

Peritoneum Peritoneum

No 199(96.6) 122(95.3) 88(89.8) 409 192(93.2) 115(89.8) 92(90.2) 399

0.485

***<0.05****

Yes 7(3.4) 6(4.7) 10(10.2) 23 14(6.8) 13(10.2) 10(9.8) 37

*p<0.05, *p<0.01, ***p<0.001, ^#^Un., unknown
