# Supplementary material for: The important role of circulating CYFRA21-1 in metastasis diagnosis and prognostic value compared with carcinoembryonic antigen and neuron-specific enolase in lung cancer patients
Source: BMC Cancer. 2017 Feb 2;17:96. doi: 10.1186/s12885-017-3070-6 (PMC5290605; doi:10.1186/s12885-017-3070-6)
Supplement: Additional file 2: Table S2. — The association analysis between CEA, NSE positive levels and ADC patients. (DOC 55 kb) [file 12885_2017_3070_MOESM2_ESM.doc]

Additional file 2: Table S2. The association analysis between CEA, NSE positive levels and ADC patients

No. (%)

**A CEA**

Neg Moderate High Total P Value

(1-10 fold) >10 fold

(n=115) (n=189) (n=141) (n=445)

**Basic Characteristics**

Age

<45 years 6(5.2) 23(12.2) 12(8.5) 41

0.135

45-60 years 40(34.8) 62(32.8) 59(41.8) 161

>60 years 69(60.0) 104(55.0) 70(49.7) 243

Sex

Male 61(53.0) 112(59.3) 75(53.2) 248

0.436

Female 54(47.0) 77(40.7) 66(46.8) 197

Stages

I+II 18(15.6) 18(9.5) 7(5.0) 43

***<0.05****

III+IV 96(83.5) 164(86.8) 128(90.8) 388

Unknown 1(0.9) 7(3.7) 6(4.2) 14

Smoke status

No 76(66.1) 105(55.6) 85(60.3) 266

0.19

Yes 39(33.9) 84 (44.4) 56(39.7) 179

**Metastasis**

Brain

No 96(83.5) 155(82.0) 115(81.6) 366

0.918

Yes 19(16.5) 34(18.0) 26(18.4) 79

Bone

No 90(78.3) 139(73.5) 89(63.1) 318

***<0.05****

Yes 25(21.7) 50(26.5) 52(36.9) 127

Liver

No 104(90.4) 177(93.7) 118(83.7) 399

***<0.05****

Yes 11(9.6) 12(6.3) 23(16.3) 46

Adrenal gland

No 110(95.7) 180(95.2) 132(93.6) 422

0.724

Yes 5(4.3) 9(4.8) 9(6.4) 23

Lymph node

No 53(46.1) 76(40.2) 54(38.3) 183

0.427

Yes 62(53.9) 113(59.8) 87(61.7) 262

Intrapulmonary

No 103(89.6) 163(86.2) 115(81.6) 381

0.183

Yes 12(10.4) 26(13.8) 26(18.4) 64

Pleural

No 100(87.0) 146(77.2) 106(75.2) 352

0.050

Yes 15(13.0) 43(22.8) 35(24.8) 93

Mediastinal

No 112(97.4) 185(97.9) 135(95.7) 432

0.508

Yes 3(2.6) 4(2.1) 6(4.3) 13

Peritoneum

No 106(92.2) 180(95.2) 126(89.4) 412

0.129

Yes 9(7.8) 9(4.8) 15(10.6) 33

*p<0.05, **p<0.001

No. (%)

**B NSE**

Neg Moderate High Total P Value

(1-2 fold) >2 fold

(n=261) (n=122) (n=62) (n=445)

**Basic Characteristics**

Age

<45 years 24(9.2) 7(5.7) 10(16.1) 41

0.175

45-60 years 99(37.9) 44(36.1) 18(29.1) 161

>60 years 138(52.9) 71(58.2) 34(54.8) 243

Sex

Male 141(54.0) 69(56.6) 38(61.3) 248

0.401

Female 120(46.0) 53(43.4) 24(38.7) 197

Stages

I+II 27(10.3) 12(9.8) 4(6.5) 43

0.842

III+IV 227(87.0) 105(86.1) 56(90.3) 388

Unknown 7(2.7) 5(4.1) 2(3.2) 14

Smoke status

No 168(64.4) 70(57.4) 28(45.2) 266

***<0.05****

Yes 93(35.6) 52 (42.6) 34(54.8) 179

**Metastasis**

Brain

No 220(84.3) 98(80.3) 48(77.4) 366

***<0.001*****

Yes 41(15.7) 24(19.7) 14(22.6) 79

Bone

No 205(78.5) 79(64.8) 34(54.8) 318

***<0.001*****

Yes 56(21.5) 43(35.2) 28(45.2) 127

Liver

No 240(92.0) 108(88.5) 51(82.3) 399

0.070

Yes 21(8.0) 14(11.5) 11(17.7) 46

Adrenal gland

No 248(95.0) 119(97.5) 55(88.7) 422

0.338

Yes 13(5.0) 3(2.5) 7(11.3) 23

Lymph node

No 117(44.8) 44(36.1) 22(35.5) 183

0.167

Yes 144(55.2) 78(63.9) 40(64.5) 262

Intrapulmonary

No 223(85.4) 108(88.5) 50(80.6) 381

0.472

Yes 38(14.6) 14(11.5) 12(19.4) 64

Pleural

No 213(81.6) 95(77.9) 44(71.0) 352

0.166

Yes 48(18.4) 27(22.1) 18(29.0) 93

Mediastinal

No 255(97.7) 117(95.9) 60(96.8) 432

0.615

Yes 6(2.3) 5(4.1) 2(3.2) 13

Peritoneum

No 242(92.7) 116(95.1) 54(87.1) 412

0.123

Yes 19(7.3) 6(4.9) 8(12.9) 33

*p<0.05, **p<0.001
