# Supplementary material for: The important role of circulating CYFRA21-1 in metastasis diagnosis and prognostic value compared with carcinoembryonic antigen and neuron-specific enolase in lung cancer patients
Source: BMC Cancer. 2017 Feb 2;17:96. doi: 10.1186/s12885-017-3070-6 (PMC5290605; doi:10.1186/s12885-017-3070-6)
Supplement: Additional file 3: Table S3. — The association analysis between bio-markers positive levels and SCC. (DOC 65 kb) [file 12885_2017_3070_MOESM3_ESM.doc]

Additional file 3: Table S3. The association analysis between bio-markers positive levels and SCC

No. (%)

**A CEA**

Neg Moderate High Total P Value

(1-10 fold) >10 fold

(n=117) (n=86) (n=12) (n=215)

**Basic Characteristics**

Age

<45 years 3(2.6) 2(2.3) 0(0.0) 5

0.883

45-60 years 41(35.0) 26(30.2) 5(41.7) 72

>60 years 73(62.4) 58(67.5) 7(58.3) 138

Sex

Male 105(89.7) 77(89.5) 10(83.3) 192

0.778

Female 12(10.3) 9(10.5) 2(16.7) 23

Stages

I+II 24(20.5) 6(7.0) 3(25.0) 33

0.056

III+IV 88(75.2) 78(90.7) 9(75.0) 175

Unknown 5(4.3) 2(2.3) 0(0.0) 7

Smoke status

No 29(24.8) 16(18.6) 2(16.7) 47

***<0.001*****

Yes 88(75.2) 70 (81.4) 10(83.3) 168

**Metastasis**

Brain

No 108(92.3) 78(90.7) 10(83.3) 196

0.569

Yes 9(7.7) 8(9.3) 2(16.7) 19

Bone

No 103(88.0) 70(81.4) 9(75.0) 182

0.188

Yes 14(12.0) 16(18.6) 3(25.0) 33

Liver

0.316

No 105(89.7) 76(88.4) 9(75.0) 190

Yes 12(10.3) 10(11.6) 3(25.0) 25

Adrenal gland

No 109(93.2) 82(95.3) 11(91.7) 202

0.766

Yes 8(6.8) 4(4.7) 1(8.3) 13

Lymph node

No 53(45.3) 24(27.9) 2(16.7) 79

***<0.05****

Yes 64(54.7) 62(72.1) 10(83.3) 136

Intrapulmonary

No 97(82.9) 77(89.5) 11(91.7) 185

0.342

Yes 20(17.1) 9(10.5) 1(8.3) 30

Pleural

No 100(85.5) 79(91.9) 9(75.0) 188

0.190

Yes 17(14.5) 7(8.1) 3(25.0) 27

Mediastinal

No 111(94.9) 82(95.3) 10(83.3) 2013

0.225

Yes 6(5.1) 4(4.7) 2(16.7) 12

Peritoneum

No 107(91.5) 83(96.5) 11(91.7) 201

0.341

Yes 10(8.5) 3(3.5) 1(8.3) 14

*p<0.05, **p<0.001

**B CYFRA**

No. (%)

Neg Moderate High Total P Value

(1-3 fold) >3 fold

(n=36) (n=85) (n=94) (n=215)

**Basic Characteristics**

Age

<45 years 1(2.8) 2(2.4) 2(2.1) 5

0.766

45-60 years 15(41.7) 25(29.4) 32(34.1) 72

>60 years 20(55.5) 58(68.2) 60(63.8) 138

Sex

Male 31(86.1) 77(90.6) 84(89.4) 192

0.767

Female 5(13.9) 8(9.4) 10(10.6) 23

Stages

I+II 11(30.6) 18(21.2) 4(4.3) 33

***<0.05****

III+IV 23(63.9) 65(76.5) 87(92.5) 175

Unknown 2(5.5) 2(2.3) 3(3.2) 7

Smoke status

No 9(25.0) 16(18.8) 22(23.4) 47

***<0.001*****

Yes 27(75.0) 69 (81.2) 72(76.6) 168

**Metastasis**

Brain

No 34(94.4) 83(97.6) 79(84.0) 196

***<0.05****

Yes 2(5.6) 2(2.4) 15(16.0) 19

Bone

No 34(94.4) 73(85.9) 75(79.8) 182

0.107

Yes 2(5.6) 12(14.1) 19(20.2) 33

Liver

No 35(97.2) 77(90.6) 78(83.0) 190

0.055

Yes 1(2.8) 8(9.4) 16(17.0) 25

Adrenal gland

No 35(97.2) 82(96.5) 85(90.4) 202

0.158

Yes 1(2.8) 3(3.5) 9(9.6) 13

Lymph node

No 21(58.3) 34(40.0) 24(25.5) 79

***<0.05****

Yes 15(41.7) 51(60.0) 70(74.5) 136

Intrapulmonary

No 30(83.3) 74(87.1) 81(86.2) 185

0.830

Yes 6(16.7) 11(12.9) 13(13.8) 30

Pleural

No 32(88.9) 78(91.8) 78(83.0) 188

0.200

Yes 4(11.1) 7(8.2) 16(17.0) 27

Mediastinal

0.524

No 34(94.4) 82(96.5) 87(92.6) 203

Yes 2(5.6) 3(3.5) 7(7.4) 12

Peritoneum

0.078

No 34(94.4) 83(97.6) 84(89.4) 201

Yes 2(5.6) 2(2.4) 10(10.6) 14

*p<0.05, **p<0.001

No. (%)

**C NSE**

Neg Moderate High Total P Value

(1-2 fold) >2 fold

(n=110) (n=70) (n=35) (n=215)

**Basic Characteristics**

Age

<45 years 3(2.7) 2(2.9) 0(0.0) 5

0.622

45-60 years 40(36.4) 23(32.8) 9(25.7) 72

>60 years 67(60.9) 45(64.3) 26(74.3) 138

Sex

Male 101(91.8) 61(87.1) 30(85.7) 192

0.463

Female 9(8.2) 9(12.9) 5(14.3) 23

Stages

I+II 26(23.6) 6(8.6) 1(2.8) 33

***<0.05****

III+IV 80(72.7) 62(88.6) 33(94.4) 175

Unknown 4(3.7) 2(2.8) 1(2.8) 7

**Metastasis**

Brain

No 107(97.3) 62(88.6) 27(77.1) 196

***<0.05****

Yes 3(2.7) 8(11.4) 8(22.9) 19

Bone

No 100(90.9) 55(78.6) 27(77.1) 182

***<0.05****

Yes 10(9.1) 15(21.4) 8(22.9) 33

Liver

No 102(92.7) 61(87.1) 27(77.1) 190

0.062

Yes 8(7.3) 9(12.9) 8(22.9) 25

Adrenal gland

No 106(96.4) 64(91.4) 32(91.4) 202

0.316

Yes 4(3.6) 6(8.6) 3(8.6) 13

Lymph node

No 51(46.4) 19(27.1) 9(25.7) 79

***<0.05****

Yes 59(53.6) 51(72.9) 26(74.3) 136

Intrapulmonary

No 96(87.3) 58(82.9) 31(88.6) 185

0.632

Yes 14(12.7) 12(17.1) 4(11.4) 30

Pleural

No 98(89.1) 59(84.3) 31(88.6) 188

0.622

Yes 12(10.9) 11(15.7) 4(11.4) 27

Mediastinal

No 108(98.2) 61(87.1) 34(97.1) 203

***<0.05****

Yes 2(1.8) 9(12.9) 1(2.9) 12

Peritoneum

No 109(99.1) 61(87.1) 31(88.6) 201

***<0.05****

Yes 1(0.9) 9(12.9) 4(11.4) 14

*p<0.05, **p<0.001
