# Supplementary material for: The important role of circulating CYFRA21-1 in metastasis diagnosis and prognostic value compared with carcinoembryonic antigen and neuron-specific enolase in lung cancer patients
Source: BMC Cancer. 2017 Feb 2;17:96. doi: 10.1186/s12885-017-3070-6 (PMC5290605; doi:10.1186/s12885-017-3070-6)
Supplement: Additional file 4: Table S4. — The association analysis between bio-markers positive levels and SCLC. (DOC 63 kb) [file 12885_2017_3070_MOESM4_ESM.doc]

Additional file 4: Table S4. The association analysis between bio-markers positive levels and SCLC

No. (%)

**A CEA**

Neg Moderate High Total P Value

(1-10 fold) >10 fold

(n= 67) (n=74) (n=18) (n=159)

**Basic Characteristics**

Age

<45 years 5(7.5) 5(6.8) 0(0.0) 10

***<0.05****

45-60 years 38(56.7) 26(35.1) 6(33.3) 70

>60 years 24(35.8) 43(58.1) 12(66.7) 79

Sex

Male 49(73.1) 65(87.8) 16(88.9) 130

0.550

Female 18(26.9) 9(12.2) 2(11.1) 29

Stages

I+II 10(14.9) 5(6.8) 0(0.0) 15

0.076

III+IV 43(64.2) 61(82.4) 15(83.3) 119

Unknown 14(20.9) 8(10.8) 3(16.7) 25

Smoke status

No 22(32.8) 20(27.1) 2(11.1) 44

0.185

Yes 45(67.2) 54 (72.9) 16(88.9) 115

**Metastasis**

Brain

No 62(92.5) 65(87.8) 17(94.4) 144

0.531

Yes 5(7.5) 9(12.2) 1(5.6) 14

Bone

0.955

No 58(86.6) 65(87.8) 16(88.9) 139

Yes 9(13.4) 9(12.2) 2(11.1) 20

Liver

0.415

No 59(88.1) 66(89.2) 14(77.8) 139

Yes 8(11.9) 8(10.8) 4(22.2) 20

Adrenal gland

No 59(88.1) 69(93.2) 17(94.4) 145

0.486

Yes 8(11.9) 5(6.8) 1(5.6) 14

Lymph node

No 23(34.3) 22(29.7) 4(22.2) 49

0.591

Yes 44(65.7) 52(70.3) 14(77.8) 110

Intrapulmonary

No 62(92.5) 70(94.6) 15(83.3) 147

0.268

Yes 5(7.5) 4(5.4) 3(16.7) 12

Pleural

No 61(91.0) 69(93.2) 17(94.4) 147

0.836

Yes 6(9.0) 5(6.8) 1(5.6) 12

Mediastinal

No 66(98.5) 72(97.3) 15(83.3) 153

***<0.05****

Yes 1(1.5) 2(2.7) 3(16.7) 6

Peritoneum

No 64(95.5) 71(95.9) 14(77.8) 149

***<0.05****

Yes 3(4.5) 3(4.1) 4(22.2) 10

*p<0.05, **p<0.001

No. (%)

**B CYFRA**

Neg Moderate High Total P Value

(1-3 fold) >3 fold

(n=62) (n=81) (n=16) (n=159)

**Basic Characteristics**

Age

<45 years 5(8.1) 4(4.9) 1(6.3) 10

0.108

45-60 years 34(54.8) 32(39.5) 4(25.0) 70

>60 years 23(37.1) 45(55.6) 11(68.7) 79

Sex

Male 47(75.8) 68(84.0) 15(93.8) 130

0.191

Female 15(24.2) 13(16.0) 1(6.3) 29

Stages

I+II 7(11.3) 8(9.9) 0(0.0) 15

0.658

III+IV 47(75.8) 59(72.8) 13(81.3) 119

Unknown 8(12.9) 14(17.3) 3(18.7) 25

Smoke status

No 23(37.1) 20(24.7) 1(6.3) 44

***<0.005****

Yes 39(62.9) 61(75.3) 15(93.7) 115

**Metastasis**

Brain

No 54(87.1) 77(95.1) 13(81.3) 144

0.110

Yes 8(12.9) 4(4.9) 3(18.7) 15

Bone

No 56(90.3) 71(87.7) 12(75.0) 139

0.256

Yes 6(9.7) 10(12.3) 4(25.0) 20

Liver

No 59(95.2) 68(84.0) 12(75.0) 139

***<0.05****

Yes 3(4.8) 13(16.0) 4(25.0) 20

Adrenal gland

0.346

No 57(92.0) 72(88.9) 16(100) 145

Yes 5(8.0) 9(11.1) 0(0.0) 14

Lymph node

No 24(38.7) 23(23.4) 2(12.5) 49

0.103

Yes 38(61.3) 58(71.6) 14(87.5) 110

Intrapulmonary

No 57(91.9) 76(93.8) 14(87.5) 147

0.668

Yes 5(8.1) 5(6.2) 2(12.5) 12

Pleural

No 59(95.2) 73(90.1) 15(93.8) 147

0.517

Yes 3(4.8) 8(9.9) 1(6.2) 12

Mediastinal

No 61(98.4) 77(95.1) 15(93.8) 153

0.504

Yes 1(1.6) 4(4.9) 1(6.2) 6

Peritoneum

0.074

No 60(96.8) 76(93.8) 13(81.2) 149

Yes 2(3.2) 5(6.2) 3(18.8) 10

*p<0.05, **p<0.001

No. (%)

**C NSE**

Neg Moderate High Total P Value

(1-2 fold) >2 fold

(n=19) (n=41) (n=99) (n=159)

**Basic Characteristics**

Age

<45 years 1(5.3) 3(7.3) 6(6.1) 10

0.431

45-60 years 12(63.2) 15(36.6) 43(43.4) 70

>60 years 6(31.5) 23(56.1) 50(50.5) 79

Sex

Male 15(79.0) 32(78.1) 83(83.8) 130

0.628

Female 4(21.0) 9(21.9) 16(16.2) 29

Stages

I+II 3(15.8) 7(17.1) 5(5.1) 15

***<0.05****

III+IV 11(57.9) 24(58.5) 84(84.8) 119

Unknown 5(26.3) 10(24.4) 10(10.1) 25

Smoke status

No 9(47.4) 12(29.3) 23(23.2) 44

***<0.001*****

Yes 10(52.6) 29 (70.7) 76(76.8) 115

**Metastasis**

Brain

No 17(89.5) 36(87.8) 91(91.9) 144

0.739

Yes 2(10.5) 5(12.2) 8(8.1) 15

Bone

No 17(89.5) 36(87.8) 86(86.9) 139

0.661

Yes 2(10.5) 5(12.2) 13(13.1) 20

Liver

No 17(89.5) 39(95.1) 83(83.8) 139

0.179

Yes 2(10.5) 2(4.9) 16(16.2) 20

Adrenal gland

No 18(94.7) 37(90.2) 90(90.9) 145

0.838

Yes 1(5.3) 4(9.8) 9(9.1) 14

Lymph node

No 11(57.9) 16(39.0) 22(22.2) 49

***<0.05****

Yes 8(42.1) 25(60.1) 77(77.8) 110

Intrapulmonary

No 19(100) 38(92.7) 90(90.9) 147

0.388

Yes 0(0.0) 3(7.3) 9(9.1) 12

Pleural

No 18(94.7) 39(95.1) 90(90.9) 147

0.638

Yes 1(5.3) 2(4.9) 9(9.1) 12

Mediastinal

No 19(100) 41(100) 93(93.9) 153

0.151

Yes 0(0.0) 0(0.0) 6(6.1) 6

Peritoneum

No 18(94.7) 40(97.6) 91(91.9) 149

0.448

Yes 1(5.3) 1(2.4) 8(8.1) 10

*p<0.05, **p<0.001
