# Supplementary material for: The important role of circulating CYFRA21-1 in metastasis diagnosis and prognostic value compared with carcinoembryonic antigen and neuron-specific enolase in lung cancer patients
Source: BMC Cancer. 2017 Feb 2;17:96. doi: 10.1186/s12885-017-3070-6 (PMC5290605; doi:10.1186/s12885-017-3070-6)
Supplement: Additional file 5: Table S5. — The association analysis between positive numbers and ADC, SCC, SCLC patients. (DOC 59 kb) [file 12885_2017_3070_MOESM5_ESM.doc]

Additional file 5: Table S5. The association analysis between positive numbers and ADC, SCC, SCLC patients

No. (%)

**A ADC**

Neg Single Double Triple Total P Value

(n=34) (n=110) (n=174) (n=127) (n=445)

**Basic Characteristics**

Age

<45 years 2(5.9) 6(5.5) 17(9.8) 16(12.6) 41

0.131

45-60 years 17(50.0) 46(41.8) 53(30.5) 45(35.4) 161

>60 years 15(44.1) 58(52.7) 104(59.7) 66(52.0) 243

Sex

Male 17(50.0) 61(55.5) 94(54.0) 76(59.8) 248

0.675

Female 17(50.0) 49(44.5) 80(46.0) 51(40.2 197

Stages

I+II 8(23.5) 18(16.4) 9(5.2) 8(6.3) 43

***<0.05****

III+IV 26(76.5) 88(80.0) 160(92.0) 114(89.8) 388

Unknown 0(0.0) 4(3.6) 5(2.8) 5(3.9) 14

Smoke status

No 23(67.6) 77(70.0) 100(57.5) 66(51.9) 266

***<0.05****

Yes 11(32.4) 33 (30.0) 74(42.5) 61(48.1) 179

**Metastasis**

Brain

No 32(94.1) 95(86.4) 137(78.7) 102(80.3) 366

0.096

Yes 2(5.9) 15(13.6) 37(21.3) 25(19.7) 79

Bone

No 32(94.1) 92(83.6) 117(67.2) 77(60.6) 318

***<0.001*****

Yes 2(5.9) 18(16.4) 57(32.8) 50(39.4) 127

Liver

0.056

No 32(94.1) 104(94.5) 156(89.7) 107(84.3) 399

Yes 2(5.9) 6(5.5) 18(10.3) 20(15.7) 46

Adrenal gland

No 33(97.0) 106(96.4) 162(93.1) 121(95.3) 422

0.576

Yes 1(3.0) 4(3.6) 12(6.9) 6(4.7) 23

Lymph node

No 23(67.6) 54(49.1) 62(35.6) 44(34.6) 183

***<0.05****

Yes 11(32.4) 56(50.9) 112(64.4) 83(65.4) 262

Intrapulmonary

No 33(97.0) 98(89.0) 142(81.6) 108(85.0) 381

0.072

Yes 1(3.0) 12(11.0) 32(18.4) 19(15.0) 64

Pleural

No 31(91.2) 94(85.5) 135(77.6) 92(72.4) 352

***<0.05****

Yes 3(8.8) 16(14.5) 39(22.4) 35(27.6) 93

Mediastinal

No 34(100) 108(98.2) 168(96.6) 122(96.0) 432

0.546

Yes 0(0.0) 2(1.8) 6(3.4) 5(4.0) 13

Peritoneum

No 34(100) 103(93.6) 159(91.4) 116(91.3) 412

0.314

Yes 0(0.0) 7(6.4) 15(8.6) 11(8.7) 33

*p<0.05, **p<0.001

No. (%)

**B SCC**

Neg Single Double Triple Total P Value

(n=19) (n=60) (n=86) (n=50) (n=215)

**Basic Characteristics**

Age

<45 years 1(5.3) 1(1.7) 2(2.3) 1(2.0) 5

0.811

45-60 years 6(36.8) 24(40.0) 27(31.4) 14(28.0) 72

>60 years 11(57.9) 35(58.30 57(66.3) 35(70.0) 138

Sex

Male 18(94.7) 54(90.0) 75(87.2) 45(90.0) 192

0.792

Female 1(5.3) 6(10.0) 11(12.8) 5(10.0) 23

Stages

I+II 8(42.1) 13(21.7) 11(12.8) 1(2.0) 33

***<0.05****

III+IV 10(52.6) 45(75.0) 71(82.6) 49(98.0) 175

Unknown 1(5.3) 2(3.3) 4(4.6) 0 7

Smoke status

No 4(21.1) 14(23.3) 20(23.3) 9(18.0) 47

0.892

Yes 15(78.9) 46(76.7) 66(76.7) 41(82.0) 168

**Metastasis**

Brain

0.086

No 19(100) 58(96.7) 76(88.4) 43(86.0) 196

Yes 0(0.0) 2(3.3) 10(11.6) 7(14.0) 19

Bone

No 18(94.7) 56(93.3) 71(82.6) 37(74.0) 182

***<0.05****

Yes 1(5.3) 4(6.7) 15(17.4) 13(26.0) 33

Liver

No 19(100) 55(91.7) 75(87.2) 41(82.0) 190

0.156

Yes 0(0.0) 5(8.3) 11(12.8) 9(18.0) 25

Adrenal gland

No 19(100) 58(96.7) 77(89.5) 48(96.0) 202

0.150

Yes 0(0.0) 2(3.3) 9(10.5) 2(4.0) 13

Lymph node

No 12(63.2) 27(45.0) 35(40.7) 5(10.0) 79

***<0.001*****

Yes 7(36.8) 33(55.0) 51(59.3) 45(90.0) 136

Intrapulmonary

0.836

No 16(84.2) 51(85.0) 73(84.9) 45(90.0) 185

Yes 3(15.8) 9(15.0) 13(15.1) 5(10.0) 30

Pleural

No 16(84.2) 57(95.0) 68(79.1) 47(94.0) 188

***<0.05****

Yes 3(15.8) 3(5.0) 18(20.9) 3(6.0) 27

Mediastinal

No 19(100) 58(96.7) 80(93.0) 46(92.0) 203

0.463

Yes 0(0.0) 2(3.3) 6(7.0) 4(8.0) 12

Peritoneum

No 19(100) 58(96.7) 77(89.5) 47(94.0) 201

0.208

Yes 0(0.0) 2(3.3) 9(10.5) 3(6.0) 14

*p<0.05, **p<0.001

No. (%)

**C SCLC**

Neg Single Double Triple Total P Value

(n=7) (n=37) (n=53) (n=62) (n=159)

**Basic Characteristics**

Age

<45 years 0(0.0) 4(10.8) 3(5.7) 3(4.8) 10

***<0.05****

45-60 years 6(85.7) 22(59.5) 22(41.5) 20(32.3) 70

>60 years 1(14.3) 11(29.7) 28(52.8) 39(62.9) 79

Sex

Male 6(85.7) 23(62.1) 47(88.7) 54(87.1) 130

***<0.05****

Female 1(14.3) 14(37.8) 6(11.3) 8(12.9) 29

Stages

I+II 1(14.3) 7(18.9) 3(5.7) 4(6.5) 15

***<0.05****

III+IV 5(71.4) 21(56.8) 44(83.0) 49(79.0) 119

Unknown 1(14.3) 9(24.3) 6(11.3) 9(14.5) 25

Smoke status

No 3(42.9) 18(48.7) 9(16.9) 14(22.6) 44

***<0.05****

Yes 4(57.1) 19(51.3) 44(83.1) 48(77.4) 115

**Metastasis**

Brain

0.317

No 6(85.7) 35(94.6) 45(84.9) 58(93.6) 144

Yes 1(14.3) 2(5.4) 8(15.1) 4(6.4) 15

Bone

No 7(100) 32(86.5) 46(86.8) 54(87.1) 139

0.786

Yes 0(0.0) 5(13.5) 7(13.2) 8(12.9) 20

Liver

No 7(100) 35(94.6) 44(83.0) 53(85.5) 139

0.156

Yes 0(0.0) 2(5.4) 9(17.0) 9(14.5) 20

Adrenal gland

No 7(100) 33(89.2) 47(88.7) 58(93.6) 145

0.635

Yes 0(0.0) 4(10.8) 6(11.3) 6(6.4) 14

Lymph node

No 6(85.7) 14(37.8) 12(22.6) 17(27.4) 49

***<0.05****

Yes 1(14.3) 23(62.1) 41(77.4) 45(72.6) 110

Intrapulmonary

0.894

No 7(100) 34(91.9) 49(92.5) 57(91.9) 147

Yes 0(0.0) 3(8.1) 4(7.5) 5(8.1) 12

Pleural

0.894

No 7(100) 34(91.9) 49(92.5) 57(91.9) 147

Yes 0(0.0) 3(8.1) 4(7.5) 5(8.1) 12

Mediastinal

No 7(100) 36(97.3) 53(100) 57(91.9) 153

0.132

Yes 0(0.0) 1(2.7) 0(0.0) 5(8.1) 6

Peritoneum

No 7(100) 35(94.6) 51(96.2) 56(90.3) 149

0.512

Yes 0(0.0) 2(5.4) 2(3.8) 6(9.7) 10

*p<0.05, **p<0.001
