# Supplementary figures and images for: The important role of circulating CYFRA21-1 in metastasis diagnosis and prognostic value compared with carcinoembryonic antigen and neuron-specific enolase in lung cancer patients
Source: BMC Cancer. 2017 Feb 2;17:96. doi: 10.1186/s12885-017-3070-6 (PMC5290605; doi:10.1186/s12885-017-3070-6)

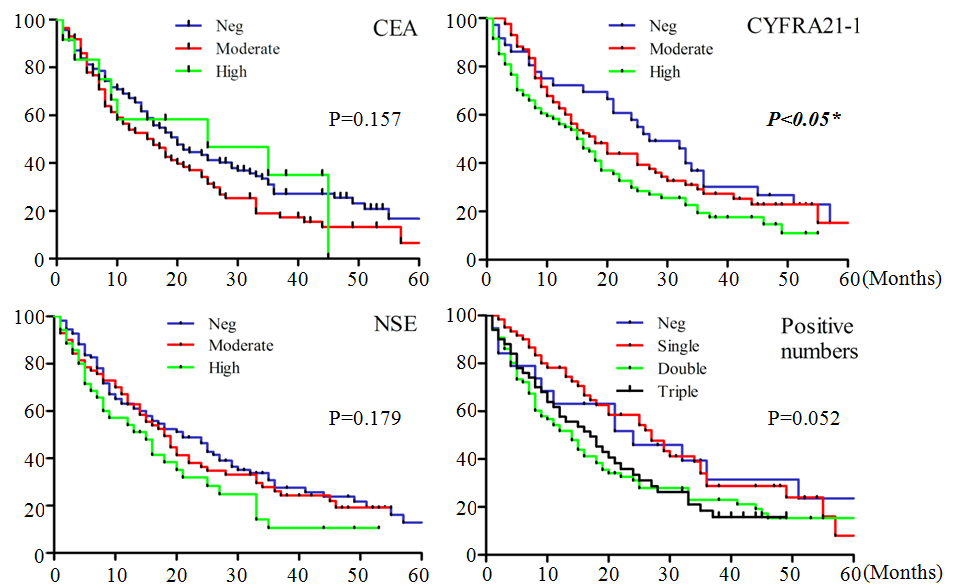

Supplement: Additional file 6: Figure S1A. — Survival functions between SCC patients and the level of CEA, CYFRA21-1, NSE and positive numbers. *P < 0.05, **P < 0.001. (TIF 1909 kb) [file 12885_2017_3070_MOESM6_ESM.tif]

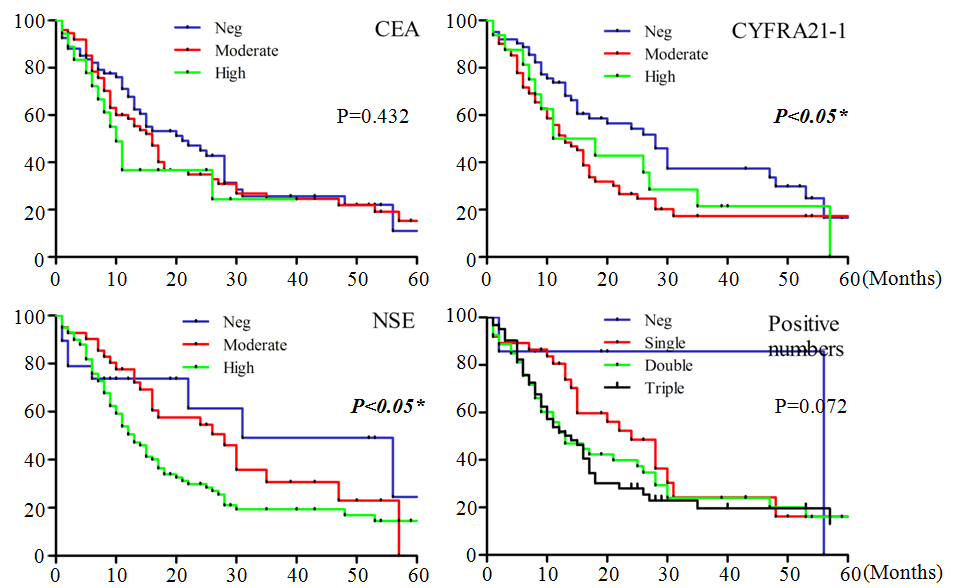

Supplement: Additional file 7: Figure S1B. — The survival functions analysis in SCLC patients based on increased concentrations in CEA, CYFRA21-1, NSE and positive numbers of biomarkers. *P < 0.05, **P < 0.001. (TIF 1899 kb) [file 12885_2017_3070_MOESM7_ESM.tif]
